# Intravenous thrombolysis before thrombectomy in acute ischemic stroke: a dual centre retrospective cohort study

**DOI:** 10.1038/s41598-022-25696-z

**Published:** 2022-12-06

**Authors:** Islam EL Malky, Mahmoud Abdelhafiz, Hazem Mo. Abdelkhalek

**Affiliations:** 1 Department of Neurology, South Valley University, Qena, Egypt; 2 Department of Neurology, Tanta University, Tanta, Egypt

**Keywords:** Neuroscience, Neurology

## Abstract

First pass effect (FPE) is a successful recanalization (mTICI ≥ 2b) after the first trial of thrombectomy. It is associated with good functional outcomes. Few studies discussed the effect of BT (bridging therapy: combined I.V. thrombolysis and mechanical thrombectomy) on FPE and clinical outcomes. In our study, we would like to report the effect of MT with or without preceding IVT on FPE and the functional outcome of AIS (Acute Ischemic Stroke) of anterior circulation in real practice. A dual-center retrospective cohort study enrolled 201 patients with AIS of anterior circulation and was divided into a bridging therapy (BT) group of 150 patients who received alteplase preceding thrombectomy, and a direct mechanical thrombectomy (dMT) group of 51 patients. Comparisons between both groups regarding the clinical and radiological outcome. Early better clinical outcome (mRS ≤ 2) at day seven with BT group (39.3%) rather than dMT (23.5%) with P value = 0.044. No significant differences as regard puncture to revascularization time, successful revascularization (mTICI) ≥ 2b and FPE between both groups (P value: 0.328, 0.538, and 0.708, respectively). No differences as regards hemorrhagic transformation, mortality rate, and 90-day favorable outcome between both groups (P value 0.091, 0.089, and 0.192, respectively). BT might have better early outcome than dMT but no difference as regards 90-day favorable outcomes, mortality, sICH, FPE, recanalization rate and procedure time. It might be reasonable to go directly to mechanical thrombectomy without IVT for AIS with large vessel occlusion.

## Introduction

First pass effect (FPE) is a successful recanalization (mTICI ≥ 2b) after the first trial of thrombectomy^1^. It is associated with good functional outcomes^2^. Few studies discussed the effect of BT (bridging therapy: combined I.V. thrombolysis and mechanical thrombectomy) on FPE and clinical outcomes^3,4^. Neurologists and Neuro-interventionalists started to ask a question about the best pathway for AIS (Acute Ischemic Stroke), if BT or dMT (direct Mechanical Thrombectomy) is more beneficial to the outcome of patients and our health care system. Some researchers thought that I.V thrombolysis (IVT) facilitate thrombectomy by softening the thrombus, and reducing the number of trials and so the procedure time^5,6^. Others reported complications in BT because of increased hemorrhagic transformation, fragmentation of the thrombus, distal embolization, and delay of thrombectomy^7,8^.

Recently, five randomized trials have investigated whether dMT resulted in comparable outcomes to BT: both DIRECT-MT and DEVT were able to demonstrate non-inferiority of MT alone paradigm, while the MR CLEAN-NO IV and SKIP results did not reach statistical significance for non-inferiority, despite comparable patient outcomes^9–12^. SWIFT-DIRECT is a RCT which found that dMT was not non-inferior to BT, with less perfusion rate^13^. DIRECT-SAFE (NCT03494920) is an ongoing one. In our study, we would like to report the effect of MT with or without preceding IVT on FPE and the functional outcome of AIS in a real practice.

## Patients and methods

### Study population

The study was carried out following local, and federal regulations and Helsinki Declaration. Written Consent from patients or their relatives was obtained to participate in this study. Dual—center retrospective cohort study was conducted for this research between July 2016 and July 2021. We included patients with AIS of LVO (large vessel occlusion) of the anterior circulation. Patients were ≥ 18 years old. Computed tomographic angiography (CTA) or magnetic resonance angiography (MRA) were used to confirm LVO. There were no restrictions based on NIHSS scale which was determined by a neurologist. An NIHSS score of 42 was considered for comatose patients with a GCS (Glasco Coma Scale) of three. Patients with modified Rankein scale (mRS) ≥ 2 prior to this stroke or posterior circulation stroke were excluded. Patients (74.6%) received intravenous thrombolytic therapy within 4.5 h, were included in bridging therapy group (BT), in accordance with American Heart Association (AHA)-American Stroke Association (ASA) recommendations^14^. If intracerebral hemorrhage had been ruled out, alteplase was given directly following the CT scan (0.9 mg/kg over 1 h with 10% of the first bolus). Included patients for thrombectomy had been selected after IV lysis failure. Patients, treated by mechanical thrombectomy without previous thrombolytic therapy, were included in direct mechanical thrombectomy group (dMT) due to missed therapeutic window for IV lysis (30 patients), oral anticoagulant (15 patients), metastatic tumors (two patients) and recent surgery or puncture (four patients). Age, sex, and vascular risk factors were all recorded as demographic data. Time estimates were made for the onset to puncture, the procedure, and the overall times (from onset to recanalization).

### Endovascular procedure

The procedure was performed with the patient under general anesthesia, using a biplane angiographic unit (Siemens Artis Zee system, Siemens Healthineers, Erlangen, Germany). All operations were performed by two senior neuro-interventionalists with more than five years of individual experience in thrombectomy of acute stroke. The femoral puncture was done by femoral sheath eight French with Seldinger technique in the common femoral artery. Then, eight French guiding catheter without balloon (Guider Soft tip, Boston Scientific) was advanced to the common carotid artery. Stent retriever (solitaire 4 mm or 6 mm, covidien or Trevo, Stryker) was used for thrombectomy inside Rebar microcatheter (Covidien). Synchro2 0.014 (Stryker) as a micro-guidewire was used. Aspiration alone was used in 20 cases (9.95%), and combined stent retrieval and aspiration in five cases (2.49%) in combination with stent retriever. Modified TICI was used to assess the degree of perfusion. Thrombectomy passes were repeated up to mTICI ≥ 2b. Angioplasty, stenting, or I.A thrombolysis were used as rescue treatment if re-occlusion or stenosis was noticed after multiple trials of thrombectomy. All radiological data was collected such as modified TICI, the number of passes (dichotomized to cases with one trial and cases with two or multiple trials), first-pass effect (mTICI ≥ 2b from the first trial), procedural complications (such as vasospasm, dissection, distal emboli, and re-occlusion). The primary efficacy outcomes were considered favorable clinical outcomes if mRS was ≤ 2 at seven days (early outcome) and after 90 days which was reported by an expert neurologist at an in-person visit or by calling. The secondary efficacy outcomes are assessed by modified Thrombolysis in Cerebral Infarction (mTICI) score where the goal is the proportion of patients who reach successful revascularization as defined by mTICI ≥ 2b at the end of the intervention. The primary safety endpoint was the mortality rate at 90 days. The secondary safety endpoint was symptomatic intracerebral hemorrhage detected on neuroimaging, 48 h after the procedure. ICH (Intracerebral hemorrhage) was classified as asymptomatic or symptomatic ICH (sICH), according to Heidelberg bleeding classification^15^. Symptomatic intracranial hemorrhage (SICH) was defined as any intracranial hemorrhage associated with ≥ 4 points increase in the NIHSS, compared to NIHSS immediately before worsening or leading to death, hemicraniotomy, or intubation.

### Statistical analysis

The collected data were statistically analyzed by software (IBM SPSS Statistics, version 25.0). We used medians, and IQR (Interquartile Range) for continuous variables, not normally distributed while mean and SD (stander deviation) for normally distributed continuous variables. Frequencies or proportions were used for categorical variables. Univariate analysis for the possible variables affecting the clinical outcome at discharge, using chi-square for categorical variables, Mann–Whitney U test, and student t-test for not normally and normally distributed continuous variables, respectively. Binary logistic regression was performed to explore the effects of IVT on clinical outcomes after adjusting for potential confounders (enter method). Hosmer–Lehmeshow was used to measure goodness-of-fit. Entered factors included those with at least marginal significance (P < 0.1) on univariate analysis and those previously reported as major affecting factors of the early functional outcome at day seven and 90 days mRS outcome**.**

### Ethical approval

The study had approval from local ethical committee of South Valley University and Tanta University. Informed consent was taken from every patient.

## Results

### Demographic and intervention results

Among 201 patients, the mean age was 64 ± 12.75 years; 102/201 (50.7%) were male. One hundred and fifty patients (74.6%) were included in BT group while 51 patients (25.4%) were in the other group (dMT). No significant difference was detected between both groups in baseline characteristics such as age, sex, vascular risk factors, sites of occlusion, and TOAST classification as reported in Table 1 with exception of shorter onset to puncture time (OPT) in BT group. The median duration of the onset to puncture time (OPT) in BT group was 4.5 h (min to max. 2.67–9.37 h) while in dMT group was 5.5 h (min to max. 2.1–21.4 h) with a statistically significant difference (P value = 0.007). The median duration of the total time (from the onset of symptoms to the end of the procedure) in BT group was 6.05 h (3.35–10.417) while in dMT group was 7.167 h (3.383–23.217) with a statistically significant difference (P value = 0.006). The mean of initial NIHSS was 14.48 ± 5.11 and 13.22 ± 5.243 in BT and dMT groups respectively without a statistical difference (P value = 0.139). The mean duration of the puncture-revascularization time in BT group was 1.389 ± 0.918 h while in dMT group was 1.516 ± 0.737 h without a statistical difference (P value = 0.328). The mean trials number was 2.7 ± 2.036 h and 3.35 ± 2.27 h in BT and dMT groups respectively without a statistical difference (P value = 0.056). Procedural complications were less in dMT group (13.7%) in comparison to BT group (19.3%) without a statistical difference (P value 0.407). Successful revascularization (mTICI) ≥ 2b was achieved in 118 patients (78.7%) in BT group, while 38 patients (74.5%) in dMT group without statistical differences (P value = 0.538). FPE was achieved in 37 patients (24.7%) in BT group, while 11 patients (21.6%) in dMT group without a statistical difference (P value = 0.708). Rescue treatment was used with 20 patients (13.3%) in BT group while nine patients (17.6%) in dMT group without a statistical difference (P value = 0.491).

**Table 1:** Baseline characters in each group.

Baseline characters	Total number (%)	BT	dMT	P value
Hypertension	61 (30.3%)	47 (13.3%)	14 (27.5%)	0.725
DM	18 (9%)	14 (9.3%)	4 (7.8%)	0.747
Smoking	33 (16.4%)	23 (15.3%)	10 (19.6%)	0.493
Hyperlipidemia	27 (13.4%)	21 (14%)	6 (11.8%)	0.814
Heart disease	24 (12%)	20 (13.4%)	4 (7.9%)	0.439
Atrial fibrillation	35 (17.4%)	29 (19.3%)	6 (11.8%)	0.286
Previous stroke	5 (2.5%)	5 (3.3%)	0 (0%)	0.228
**Site of occlusion**				
MCA(M1)	113 (56.2%)	80 (53.3%)	33 (64.7%)	0.466
MCA (M2,3)	26 (12.9%)	21 (14%)	5 (9.8%)	
ICA	30 (14.9%)	24 (16%)	6 (11.8%)	
Tandem lesion	32 (15.7%)	25 (16.7%)	7 (13.7%)	
**TOAST**				
Large vessel occlusion	33 (16.4%)	29 (19.3%)	4 (7.8%)	0.243
Cardioembolic	93 (46.3%)	65 (43.3%)	28 (54.9%)	
Other determined etiology	15 (7.5%)	11 (7.3%)	4 (7.4%)	
Undetermined or mixed etiology	60 (29.9%)	45 (30%)	15 (29.4%)	

*BT* bridging therapy, *dMT* direct mechanical thrombectomy.

### Clinical outcome

Fifty-nine patients (39.3%) had favorable clinical outcome (mRS ≤ 2) at 7 days early outcome in BT group while 12 patients (23.5%) in dMT with statistical difference (P value = 0.044). Eighty-five patients (56.7%) had 90-day favorable outcome (mRS ≤ 2) in BT group while 23 patients (45.1%) in dMT without statistical difference (P value = 0.192). Twenty-nine patients (19.3%) had procedural complications in BT group while seven patients (13.7%) in dMT group without statistical difference (P value = 0.407). Symptomatic ICH (sICH) was reported 48 h after the procedure in 11 patients (7.3%) in BT group while eight patients (15.7%) in dMT group without statistical difference (P value = 0.097). Mortality rate in BT group was 19 patients (12.7%) while 11 patients (21.6%) in dMT group without statistical difference (P value = 0.17) after the procedure. Total mortality rate at 90 days in BT group was 22 patients (14.7%) while 13 patients (25.5%) in dMT group without statistical difference (P value = 0.089). Table 2 reported multiple variables, analyzed by binary logistic regression after univariate analysis for the possible effect on the early clinical outcome at day seven in addition to intravenous thrombolysis such as recanalization rate, total time and NIHSS at admission Table 3 reported that NIHSS and recanalization rate were the only predictors of 90 days favorable outcome.

**Table 2:** Multivariate logistic regression of variables affecting good clinical outcome at day seven.

Predictor of good outcome	P value univariate analysis	P value of OR after LOGISTIC regression^a^	Adjusted OR (95% CI)
I.V thrombolysis	0.044	0.020*	2.7 (1.19–6.67)
High recanalization rate	0.005	0.005*	3.68 (1.517–9.804)
Low NIHSS at admission	0.000	0.000*	0.809 (0.748–0.874)
Total time	0.01	0.224	0.999 (0.996–1.002)

*OR* odds ratio, *CI* confidence intervals, *OPT* onset-puncture time.

*Means statistically significant.

^a^Goodness-of-fit was measured by Hosmer–Lehmeshow parameter (P value > 0.05).

**Table 3:** Multivariate logistic regression of variables affecting good 90 days clinical outcome.

Predictors of good outcome	P value univariate analysis	P value of OR after logistic regression^a^	Adjusted OR (95% CI)
I.V thrombolysis	0.193	0.087	1.912 (4.032–0.911)
High recanalization rate	0.005*	0.005*	3.011 (1.401–6.410)
Low NIHSS at admission	0.000*	0.000*	0.854 (0.797–0.916)
Total time	0.105	0.500	0.994 (0.987–1.001)

*OR* odds ratio, *CI* confidence intervals.

*Means statistically significant.

^a^Goodness-of-fit was measured by Hosmer–Lehmeshow parameter (P value > 0.05).

## Discussion

Our dual-center retrospective real-world study reported that BT group had a better early favorable outcome (at day seven with a statistical difference but there was no statistical difference between both groups (BT, and dMT) as regards 90-day favorable outcome, sICH, and mortality. This may be due to the approved limited ability of tPA on early recanalization in LVO stroke^16^. There was no statistical difference as regard recanalization rate, FPE, and puncture-revascularization time between both groups despite significantly shorter OPT in BT group because most of the patients in the dMT group presented to the emergency room late (outside the 4.5 h window). Similar results from other retrospective and real practice studies were reported with shorter OPT in BT group and similar baseline characters in both groups^17,18^. This could represent a theoretical advantage for better outcomes in BT group, however, our analysis does not confirm the effect of OPT in early (at day seven) or late (90-day) favorable outcomes (Tables 2, 3). Two trials DIFFUSE and DAWN confirmed the tissue window paradigm, more than the time window paradigm, going in line with our study which approved the effect of initial NIHSS at admission on early and late clinical outcome^19,20^. NIHSS is well known to be a mirror of tissue danger severity. Multiple meta-analyses reported the same clinical and radiological results^21,22^. One of them included three RCTs and nine observational studies^21^. The analysis of the only three RCTs reported no statistically significant differences for a 90-day favorable outcome, mortality, successful recanalization, and sICH. A prospective cohort (ANGET-ACT registry) represented a real practice of stroke treatment and compared BT against dMT in Chinese people^23^. This study reported no difference between both groups as regards clinical and radiological outcomes but dMT had a lower risk of sICH. Zhang et al. reported in another metanalysis the same result in addition to less rate of sICH and clot migration in dMT group^24^. Jang et al. in a meta-analysis reported that FPE was associated with favorable 90-day outcomes with less morbidity without any effect of IVT between cases with FPE or without^3^. Blair et al. reported in a retrospective study that patients with FPE who received IVT had no benefit as regards clinical outcome in comparison with patients with multiple passes and received IVT^4^. That might be due to the thrombolysis effect of alteplase on distal infarctions due to fragmentation of the thrombus after multiple trials of thrombectomy.

Li et al. reported in a meta-analysis a better 90-day favorable outcome in BT than dMT which is contradictory to our study^25^. Also, the Italian Registry of Endovascular Stroke Treatment, as a real practice study, reported that BT had less sever functional dependence and mortality in three months^26^. Multiple factors might be present, leading to these contradictory results. First of all, different OPT was present between both groups in these studies, in addition to different sites (even posterior circulation territories were involved) and causes of the stroke. One of the main advantages of IVT is early recanalization, which is related to the occlusion site and length of the thrombus^27^. Whereas ICA (Internal Carotid artery) and tandem occlusions are less likely to benefit from IVT, distal middle cerebral artery occlusions seem to achieve more favorable outcomes with alteplase^28^. Second, treatment options might be different between the studies as regards the dose of alteplase (0.9 mg/kg or 0.6 mg/kg). Third, the mother ship might differ from drip and ship, because the second transfer modality gives a longer time for alteplase to be in contact with the thrombus leading to the dissolving of the thrombus or becoming less attached to the vascular wall.

Our study has some limitations. First, it is a retrospective study, without randomization of cases on both groups. Second: in spite of similar baseline characteristics in both groups, OPT was different between both groups and dMT group included cases with late-onset and ineligible for IVT injection. We recommend being adherent to the guidelines by giving IVT within the time window of 4.5 h until the appearance of more RCTs might avoid the previous caveats in older ones.

## Conclusion

BT might have better early outcome than dMT but no difference as regards 90-day favorable outcomes, mortality, sICH, FPE, recanalization rate and procedure time. It might be reasonable to go direct to mechanical thrombectomy without IVT for AIS with large vessel occlusion.

## Data Availability

The data will be available with the corresponding author at demand.

## References

[1] Zaidat OO (2018). First pass effect: A new measure for stroke thrombectomy devices. Stroke.

[2] Bai X (2021). Influence of first-pass effect on recanalization outcomes in the era of mechanical thrombectomy: A systemic review and meta-analysis. Neuroradiology.

[3] Jang KM, Choi HH, Nam TK, Byun JS (2021). Clinical outcomes of first-pass effect after mechanical thrombectomy for acute ischemic stroke: A systematic review and meta-analysis. Clin. Neurol. Neurosurg..

[4] Blair C (2021). Intravenous thrombolysis is associated with less disabling stroke and lower mortality in multiple-pass endovascular thrombectomy. Cerebrovasc. Dis..

[5] Guedin P (2015). Prior IV thrombolysis facilitates mechanical thrombectomy in acute ischemic stroke. J. Stroke Cerebrovasc. Dis..

[6] Mueller L (2017). Impact of intravenous thrombolysis on recanalization rates in patients with stroke treated with bridging therapy. Eur. J. Neurol..

[7] Tsivgoulis G (2017). Endovascular thrombectomy with or without systemic thrombolysis?. Ther. Adv. Neurol. Disord..

[8] Balodis A (2019). Endovascular thrombectomy in anterior circulation stroke and clinical value of bridging with intravenous thrombolysis. Acta Radiol..

[9] Yang P (2020). Endovascular thrombectomy with or without intravenous alteplase in acute stroke. N. Engl. J. Med..

[10] Zi W (2021). Effect of endovascular treatment alone vs intravenous alteplase plus endovascular treatment on functional independence in patients with acute ischemic stroke: The DEVT randomized clinical trial. JAMA.

[11] LeCouffe NE (2021). A randomized trial of intravenous alteplase before endovascular treatment for stroke. N. Engl. J. Med..

[12] Suzuki K (2021). Effect of mechanical thrombectomy without vs with intravenous thrombolysis on functional outcome among patients with acute ischemic stroke: The SKIP randomized clinical trial. JAMA.

[13] Fischer URS (2022). Thrombectomy alone versus intravenous alteplase plus thrombectomy in patients with stroke: An open-label, blinded-outcome, randomised non-inferiority trial. Lancet.

[14] Powers WJ (2019). Guidelines for the early management of patients with acute ischemic stroke: 2019 update to the 2018 guidelines for the early management of acute ischemic stroke: A guideline for healthcare professionals from the American Heart Association/American Stroke Association. Stroke.

[15] von Kummer R (2015). The Heidelberg bleeding classification: Classification of bleeding events after ischemic stroke and reperfusion therapy. Stroke.

[16] Candel CS (2019). Recanalization of emergent large intracranial vessel occlusion through intravenous thrombolysis: Frequency, clinical outcome, and reperfusion pattern. Cerebrovasc. Dis..

[17] Machado M (2021). Functional outcome after mechanical thrombectomy with or without previous thrombolysis. J. Stroke Cerebrovasc. Dis..

[18] Rocha MG (2019). Primary thrombectomy versus combined mechanical thrombectomy and intravenous thrombolysis in large vessel occlusion acute ischemic stroke. J. Stroke Cerebrovasc. Dis..

[19] Albers GW (2018). Thrombectomy for stroke at 6 to 16 hours with selection by perfusion imaging. N. Engl. J. Med..

[20] Nogueira RG (2018). Thrombectomy 6 to 24 hours after stroke with a mismatch between deficit and infarct. N. Engl. J. Med..

[21] Du H (2021). Intravenous thrombolysis before mechanical thrombectomy for acute ischemic stroke: A meta-analysis. J. Am. Heart Assoc..

[22] Podlasek A, Dhillon PS, Butt W, Grunwald IQ, England TJ (2021). Direct mechanical thrombectomy without intravenous thrombolysis versus bridging therapy for acute ischemic stroke: A meta-analysis of randomized controlled trials. Int. J. Stroke.

[23] Tong X (2021). Thrombectomy versus combined thrombolysis and thrombectomy in patients with acute stroke: A matched-control study. Stroke.

[24] Zhang J (2022). Direct endovascular treatment versus bridging therapy in patients with acute ischemic stroke eligible for intravenous thrombolysis: Systematic review and meta-analysis. J. NeuroInterventional Surg..

[25] Li S (2021). Endovascular treatment with and without intravenous thrombolysis in large vessel occlusions stroke: A systematic review and meta-analysis. Front. Neurol..

[26] Casetta I (2019). Combined intravenous and endovascular treatment versus primary mechanical thrombectomy. The Italian registry of endovascular treatment in acute stroke. Int. J. Stroke.

[27] Saqqur M (2007). Site of arterial occlusion identified by transcranial Doppler predicts the response to intravenous thrombolysis for stroke. Stroke.

[28] Rohan V (2014). Length of occlusion predicts recanalization and outcome after intravenous thrombolysis in middle cerebral artery stroke. Stroke.

